# Genome-wide association analysis reveals QTL and candidate mutations involved in white spotting in cattle

**DOI:** 10.1186/s12711-019-0506-2

**Published:** 2019-11-08

**Authors:** Swati Jivanji, Gemma Worth, Thomas J. Lopdell, Anna Yeates, Christine Couldrey, Edwardo Reynolds, Kathryn Tiplady, Lorna McNaughton, Thomas J. J. Johnson, Stephen R. Davis, Bevin Harris, Richard Spelman, Russell G. Snell, Dorian Garrick, Mathew D. Littlejohn

**Affiliations:** 1 Massey University Manawatu, Private Bag 11 222, Palmerston North, 4442 New Zealand; 2 Livestock Improvement Corporation (LIC), 605 Ruakura Rd, Newstead, 3286 New Zealand; 3 The University of Auckland, Private Bag 92019, Auckland, 1142 New Zealand

## Abstract

**Background:**

White spotting of the coat is a characteristic trait of various domestic species including cattle and other mammals. It is a hallmark of Holstein–Friesian cattle, and several previous studies have detected genetic loci with major effects for white spotting in animals with Holstein–Friesian ancestry. Here, our aim was to better understand the underlying genetic and molecular mechanisms of white spotting, by conducting the largest mapping study for this trait in cattle, to date.

**Results:**

Using imputed whole-genome sequence data, we conducted a genome-wide association analysis in 2973 mixed-breed cows and bulls. Highly significant quantitative trait loci (QTL) were found on chromosomes 6 and 22, highlighting the well-established coat color genes *KIT* and *MITF* as likely responsible for these effects. These results are in broad agreement with previous studies, although we also report a third significant QTL on chromosome 2 that appears to be novel. This signal maps immediately adjacent to the *PAX3* gene, which encodes a known transcription factor that controls *MITF* expression and is the causal locus for white spotting in horses. More detailed examination of these loci revealed a candidate causal mutation in *PAX3* (p.Thr424Met), and another candidate mutation (rs209784468) within a conserved element in intron 2 of *MITF* transcripts expressed in the skin. These analyses also revealed a mechanistic ambiguity at the chromosome 6 locus, where highly dispersed association signals suggested multiple or multiallelic QTL involving *KIT* and/or other genes in this region.

**Conclusions:**

Our findings extend those of previous studies that reported *KIT* as a likely causal gene for white spotting, and report novel associations between candidate causal mutations in both the *MITF* and *PAX3* genes. The sizes of the effects of these QTL are substantial, and could be used to select animals with darker, or conversely whiter, coats depending on the desired characteristics.

## Background

Coat patterning traits provide visual characteristics that allow differentiation between domesticated animal breeds and between strains within breeds. White spotting is one of these phenotypes, and is a feature of a variety of mammals including cattle, horses, dogs, cats and mice. White spotting is a complex quantitative trait, for which several genes with major effects have been described and are relevant across species, as well as many other loci with small effects that account for the remaining genetic variance [1]. This oligogenic architecture derives from the multifaceted biology that contributes to white spotting of the coat, which is hypothesised to arise from abnormal melanocyte precursor migration and/or development. Mouse models have demonstrated that pigment cells originate from the neural crest cells via the SOX10 positive glial bipotent progenitor cells during embryogenesis, and migrate dorsally via the neural tube [2]. These cells proceed to differentiate into melanoblasts by acquiring expression of the genes *micropthalmia*-*associated transcription factor* (*MITF*), *proto*-*oncogene receptor tyrosine kinase* (*KIT*) and *dopachrome tautomerase* (*DCT*), and migrate down the ventral axis of the body. When the cells reach their destination, they migrate into the epidermis where some melanoblasts localise to the hair follicle and differentiate into melanocytes. A subset of melanoblasts dedifferentiate, losing *MITF* and *KIT* gene expression, and colonise the hair follicle bulge where they act as melanocyte stem cells and replenish differentiated melanocytes during subsequent hair cycles [2]. Disruption of any of the above processes is expected to result in parts of the body lacking mature melanocytes, and thus regions of abnormal pigmentation in the hair coat.

Quantitative trait loci (QTL) and mutations that cause white spotting have been described for a variety of species. Genetic studies in the horse revealed an inversion in the *KIT* gene associated with the Tobiano white-spotting [3], and a mutation in the *PAX3* gene associated with a splashed white pattern [4, 5]. Several mutations in the *KIT* gene have also been associated with complete white [6] or roan coat phenotypes [7]. Studies on white spotting in dogs have revealed associations with the *MITF* gene [8], and in mice more than 10 genes have been reported to be associated with white spotting traits, including the *KIT* and *MITF* genes [9]. Comparatively few studies have investigated the genetics of white spotting in cattle. Liu et al. [10] found significant QTL on chromosomes 6, 18 and 22 using linkage analysis within Holstein–Friesian (HF) × Jersey (J) crossbred cows. It has been suggested that the QTL on chromosomes 6 and 22 might be underpinned by the *KIT* and *MITF* genes, respectively [10]. Fontanesi et al. [11] compared the sequences of the *MITF* gene in white spotted Italian Holstein and Simmental cattle, and solid coloured Italian Brown and Reggiana cattle, and found a haplotype (carrying allele g.31831615T) that is associated with white spotting. This haplotype accounts for some, but not all of the variation observed in the white spotting phenotype [11]. More recently, Hofstetter et al. [12] investigated atypical white spotting in Brown Swiss cattle. They identified two completely linked single nucleotide variants within the 5′ regulatory region of the *MITF* gene associated with white spotting, and although these variants largely account for the manifestation of white spotting, they do not account for the variability between individuals, which provides further evidence for a polygenic trait [12]. Hayes et al. [1] detected the *MITF* and *KIT* genes in a genome-wide association study (GWAS) that investigated the proportion of black in black and white Holstein cows, and reported an additional signal on chromosome 8, which carries *PAX5* i.e. another potential candidate gene for this trait [1]. Together these studies converge on the involvement of *KIT* and *MITF* gene expression in white spotting in dairy cattle, however the causal variants that drive these effects have yet to be definitively identified and may be breed-specific.

Here, our aim was to investigate white spotting in New Zealand dairy cattle, by using whole-genome sequence genotype data to conduct the largest GWAS of white spotting to date. We report three genome-wide significant QTL for white spotting. Effects on chromosomes 6 and 22 extend on previous associations at these loci, and further implicate the *KIT* and *MITF* genes as responsible for these effects. For the first time, we also report a QTL on chromosome 2 that implicates the *PAX3* gene in white spotting of dairy cattle and highlight an amino acid substitution that may underlie this effect.

## Methods

### Study population

White spotting data were derived from several cohorts of animals that included: 885 outbred dairy bulls (223 J, 327 HF, and 335 HF × J), 1389 outbred dairy cows (51 J, 265 HF, and 1073 HF × J), and 699 HF × J F2 cross cows from an experimental pedigree. Breed definitions, in these cases, define animals from a 4-generation pedigree that were ^16^/_16_ J or HF as purebreds, with ^15^/_16_ animals defined as crossbreeds. The F2 animals were ½ HF × ½ J, representing a study population that was previously described in several publications [10, 13–15]. Genotyping data were available for 2973 animals, with genotype and phenotype information derived as described in the following sections.

### Measurements of white spotting in our study population

For animals in the F2 population, proportion of white spotting values that had been derived for a previous study [10] were used directly in the current study. Video footage was recorded on 1389 cows walking single file either into or out of the milking shed using a GoPro HERO4 camera, at a 4000 pixel horizontal resolution. Still images that provide a clear side-on view of each animal were captured from the video footage using VideoPad Video Editor (v5.3). Additional side-on images representing either the right or left profile of 885 bulls were made available by LIC and incorporated into the dataset. First, cows and bulls were scored for the presence or absence of white on their coat and, then, the proportion of white spotting was quantified. Quantification was carried out manually using the image processing software, GNU Image Manipulation Program (GIMP, v2.9.8), to generate an objective measurement of the proportion of white color. The freehand tool was used to trace each animal and remove the background. The pixel count from the remaining image, and the pixel count after manually subtracting the white regions on the coat, were used to calculate the proportion of white spotting on the coat.

### Genotypes, whole-genome sequencing, and sequence imputation

For 760 of the outbred cows included in the study, tissue samples were obtained from ear tissue biopsies and DNA extraction and genotyping were performed by GeneSeek (Lincoln, NE, USA) using the GeneSeek GGP50 k SNP chip. For all the remaining individuals, we used available single nucleotide polymorphism (SNP) genotypes that were previously obtained by genotyping at Geneseek on a variety of platforms including the Geneseek GGPv1, GGPv2, GGPv3, GGP50 k, Illumina BovineSNP50 or BovineHD 777k SNP chips. A full list of the genotyping platforms, the number of SNPs per panel and the number of animals genotyped per panel are in Additional file 1: Table S1. Subsets of the reference and target populations that are described in this paper have been published by Lopdell et al. [16], and Littlejohn et al. [14, 17].

Whole-genome sequencing, read mapping, and variant calling were performed on a population of 116 HF, 95 J and 354 crossbred cattle as previously described [16, 17]. Briefly, DNA samples were sequenced based on 100-bp paired-end reads on the Illumina Hiseq platform, read mapping was performed using the UMD3.1 genome build and the BWA MEM 0.7.8 software [18] and resulted in mean and median mapped read depths of 15× and 8×, respectively. Variants were called using the GATK HaplotypeCaller (v3.2) software [19], which incorporates base quality score recalibration. Then, phasing of the variants was performed using Beagle 4 [20], and variants with phasing allelic R^2^ metrics lower than 0.95 were excluded for quality filtering purposes. These criteria yielded the ~ 19.5 M whole-genome sequence variants that constituted the reference set for imputation into the 2973 SNP-chip genotyped samples used for GWAS.

A step-wise imputation was performed using the Beagle 4 software [20]. Note that these procedures were conducted to create an imputed sequence resource that is much larger than that used in the current study and represented ~ 150,000 animals, which have been accumulated over time and imputed in three different batches. The overall pipeline was as follows: first, the animals that were typed on the GGP panels were imputed to a reference panel representing the BovineSNP50 SNP-chip. Then, BovineSNP50 data (now consisting of both imputed and physically genotyped data) were used to impute all the animals to the BovineHD platform. We also conducted a parallel step to impute all the samples to the GGPv3 platform, to recover non-overlapping content between that platform and the BovineSNP50 SNP-chip. These steps yielded two datasets that comprised an ‘all animals imputed to BovineHD’ set, and an ‘all animals imputed to GGPv3′ set. These datasets were then merged, creating a scaffold for genome sequence imputation that contained all the animals imputed to all content from all SNP-chips. Following sequence imputation (by using Beagle 4), data were then filtered to remove variants with extreme Hardy–Weinberg statistics (HW exact test; removal of 47,660 variants based on *p* < 1 × 10^−30^), and near-monomorphic positions (minor allele frequency (MAF) < 0.0001; removal of 911,633 variants). These criteria yielded 18,641,995 variants, which were extracted for the subset of 2973 animals with color phenotypes from the larger ~ 150,000 animal dataset. In terms of genetic representativeness between the sequence reference animals and the 2973 GWAS animals, 1282 cattle were directly represented by both a sequenced sire and maternal grandsire in the reference dataset, of which 1122 were represented by a sire or maternal grandsire in this population.

### Population structure adjustments, covariates, and GWAS

To address population stratification in the association models due to breed and relatedness, genomic relationship matrices (GRM) were generated using GCTA (v1.91.1 beta). These calculations involved the creation of 29 GRM, one for each bovine autosome, to enable a ‘leave one chromosome out’ GWAS approach where each GRM differs by the absence of a single autosome—thus avoiding double fitting when testing the effect of candidate variants. These GRM were calculated using a curated subset of variants from the Illumina BovineSNP50 platform, which comprised 34,963 variants that had been quality-filtered based on Mendelian concordance parameters, minor allele frequency (those with a MAF < 0.02 were removed), LD pruning (those with a R^2^ > 0.9 were removed), and deviation from Hardy–Weinberg equilibrium (those with a p < 0.15 were removed). The GCTA (v1.91.1 beta) software was used to conduct the mixed linear model-based association analysis (MLMA), which incorporates the GRM as outlined above, in addition to fixed effects for farm of origin and cohort (the latter relevant to the F2 animals with the first cohort born in spring 2000 and the second cohort born in spring 2001 [13–15, 17, 21]). Whole-genome sequence variants were filtered to remove the variants with a MAF lower than 0.005 prior to MLMA, this filter being different to that applied previously based on the frequencies present in the subpopulation of 2973 animals. To account for multiple hypothesis testing, a *p* value threshold of 5 × 10^−8^ was deemed to be significant for variant associations.

### Visualization and interpretation of association results and candidate variants

To assess candidacy of the associated variants, RNA-seq data representing black and white bovine skin were sourced from a data submission accompanying the Koufariotis et al. [22] paper, and uploaded into the Integrative Genomics Viewer (IGV) for visualization [23]. Sequence variants in intervals of interest were functionally annotated by using SNPEff (v4.3) [24] and the Ensembl UMD3.1 gene annotation set, with custom scripts to visualize these effects in Manhattan plots. To assess conservation metrics for candidate causal variants, genome evolutionary rate profiling (GERP) scores were obtained for the 32-way amniota vertebrae alignments (v92.31) from the Ensembl portal, with both element and site-wise scores reported in the text [25, 26]. For multiple protein alignments that were used to investigate the conservation of the *PAX3* p.Thr424Met mutation, *PAX3* homologues were retrieved for other species using BLAST, and aligned using the Geneious software [27].

### Structural variant analysis

Sequence alignments representing the three major QTL regions were manually inspected in animals that displayed segregating tag-SNP genotypes to detect gene-disrupting structural mutations that might explain these QTL. However, given the ambiguity of the association signals at the chromosome 6 locus, a more formal analysis was conducted. Here, CNVnator (v0.3.3) [28] was used to predict the presence of structural variants based on sequence read depth, using the same whole-genome sequence dataset as described in the ‘Genotypes, whole-genome sequencing, and sequence imputation’ section. This analysis used a sliding window size of 1000-bp with a 500-bp overlap and focused on a 20-Mb region on chromosome 6 (60 to 80 Mb). Then, predicted structural variants were ranked based on their genotype correlation with the top two QTL tag variants at the chromosome 6 locus (Chr6 g.64210286A>G rs451683615 and Chr6 g.71722665C>T rs463810013). Sequence alignments of relevant variants were visually inspected in IGV [23] to assess evidence of a legitimate structural variant at each of these sites, weighted in the context of read mapping quality, gaps and/or other issues with the reference genome assembly, and whether the variant was polymorphic between samples. CNVnator-assigned genotypes were assessed in the same way for multimodality by visual inspection of copy number histograms.

## Results

Since white spotting might be influenced by genes that operate via different mechanisms, we conducted two separate GWAS that differed in the definition of the phenotype. First, white spotting was scored as the presence or absence of white on the coat and encoded as a binary phenotype (N = 2973 animals). Second, white spotting was coded as a quantitative variable, where animals were scored based on the overall proportion of white (N = 2232 animals). Solid color animals were not included in the latter population, for which proportion of white was also log-transformed prior to association analysis to render data in a form approximating a normal distribution. All phenotypic measures were based on manual analysis of photographs (see Methods section), that included images representing 699 Holstein–Friesian × Jersey (HF × J) F2 cows scored as part of a previous QTL study [10]. The breed composition and sexes of the remaining animals are described in the Methods section, which include a mixture of HF, J, and HF × J cows and bulls.

**Fig. 1:**
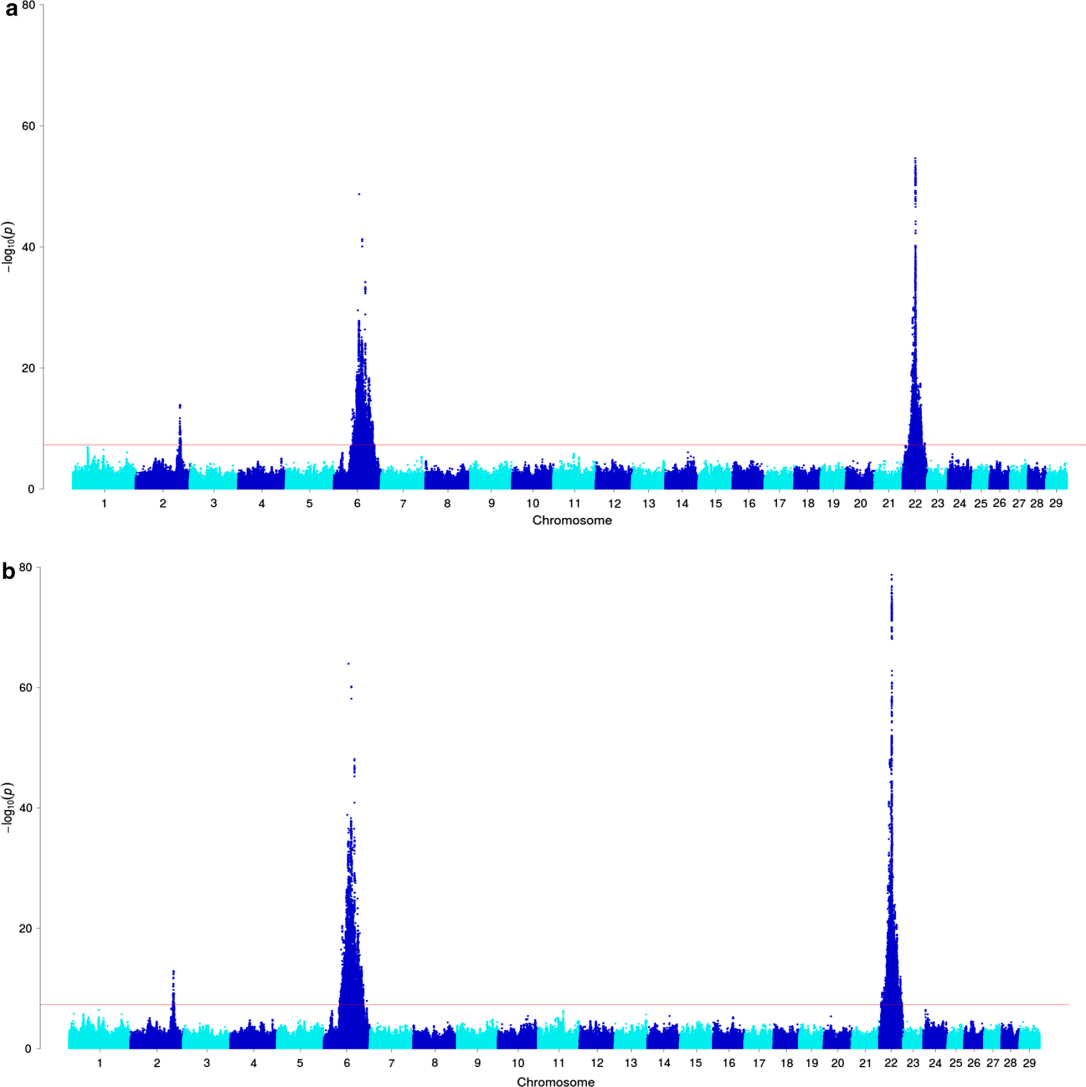
**a** Manhattan plot based on the GWAS results for the presence/absence of white color on the coat. The top variants on chromosome 22, 6 and 2 have p-values of 1.51 × 10^−56^, 3.73 × 10^−53^ and 3.26 × 10^−15^, respectively. **b** Manhattan plot based on the GWAS results for the proportion of white spotting. The top variants on chromosome 22, 6 and 2 have p-values of 1.83 × 10^−79^, 1.1 × 10^−64^ and 1.27 × 10^−13^, respectively. The red line indicates the genome-wide significance threshold *p* = 5 × 10^−8^

Genome-wide association analysis was conducted based on imputed whole-genome sequence genotypes using the GCTA (v1.91.6) software. Genotypes were imputed to sequence resolution using a reference population of 565 whole-genome-sequenced animals and methods that are similar to those described previously (see “Methods” section and Lopdell et al. [16]). The mixed linear models assumed additivity and incorporated adjustments for farm of origin, cohort [10], and a genomic relationship matrix (GRM) computed in GCTA (v1.91.6). Imputed data were also filtered to remove variants that had a MAF lower than 0.005 and met other quality filtering criteria described in more detail in the Methods section. Results of the association analysis for presence/absence of white on the coat revealed three signals that surpassed the genome-wide significance threshold of *p* = 5 × 10^−8^ and were located on chromosomes 2, 6, and 22 (Fig. 1a). The top variants for these QTL mapped to Chr 22 g.31769747A>G (rs209784468, *p* = 1.51 × 10^−56^), Chr 6 g.64210286A>G (rs451683615, *p* = 3.73 × 10^−53^), and Chr 2 g.111576221A>C (rs109979909, *p* = 3.26 × 10^−15^).

For the analysis that treated white spotting as a quantitative variable (proportion of white spotting), GWAS revealed the same three regions as those described for the binary-encoded trait (*p* < 5 × 10^−8^; Fig. 1b). Furthermore, this analysis presented the same three top-associated variants that were identified in the first GWAS, which suggested that these signals represented the same QTL. These results are in agreement with previous findings that described white spotting as a quantitative trait, i.e. under the control of multiple QTL [1, 11]. Given that the signals derived from the quantitative phenotype were also more significant than for the binary trait, this phenotype became the focus of the analyses that are presented below. Table 1 lists the top 10 associated variants and the effects of these QTL. Notably, the sizes of the effects of all three QTL were very large, with allele substitution effects of 3.2, 12.9, and 11.5% for the top tag SNPs on chromosomes 2, 6, and 22, respectively.

**Table 1:** Top 10 variants for each significant quantitative trait locus detected in the genome-wide association analysis for proportion of white spotting

Variant reference ID		Genomic position	Effect size (%)^a^	Standard error	p-value
*Chromosome 22*					
1	rs209784468	Chr22 g.31769747A>G	11.52	0.129	1.83 × 10^−79^
2	rs461193589	Chr22 g.31783093T>C	11.35	0.129	8.67 × 10^−79^
3	rs456585934	Chr22 g.31888569A>G	11.43	0.13	1.21 × 10^−78^
4	rs209274730	Chr22 g.32386542A>C	11.06	0.129	1.38 × 10^−77^
5	rs480312583	Chr22 g.31958551G>A	11.2	0.13	2.47 × 10^−77^
6	NA	Chr22 g.31768931A>T	10.88	0.129	6.11 × 10^−77^
7	rs208958980	Chr22 g.31769772T>C	10.84	0.129	1.59 × 10^−76^
8	rs433645096	Chr22 g.31768933A>T	10.84	0.129	1.59 × 10^−76^
9	NA	Chr22 g.31768928TG>T	10.82	0.129	2.29 × 10^−76^
10	rs209837244	Chr22 g.32369667G>A	10.94	0.129	2.57 × 10^−76^
*Chromosome 6*					
1	rs451683615	Chr6 g.64210286A>G	12.86	0.15	1.10 × 10^−64^
2	rs463810013	Chr6 g.71722665C>T	12.27	0.152	6.37 × 10^−61^
3	rs109512689	Chr6 g.71873479T>C	12.02	0.151	8.08 × 10^−61^
4	rs385773341	Chr6 g.71873455A>C	12.02	0.151	8.08 × 10^−61^
5	rs474403670	Chr6 g.71698814A>G	12.22	0.152	8.99 × 10^−61^
6	rs208251862	Chr6 g.71692344C>A	10.62	0.146	7.05 × 10^−59^
7	rs43469863	Chr6 g.79629052T>C	7.76	0.139	7.47 × 10^−49^
8	rs43469866	Chr6 g.79631054T>C	7.69	0.139	1.34 × 10^−48^
9	rs43764915	Chr6 g.79649488A>G	7.54	0.139	9.51 × 10^−48^
10	rs208257925	Chr6 g.79640038G>A	7.48	0.139	1.90 × 10^−47^
*Chromosome 2*					
1	rs109979909	Chr2 g.111576221A>C	3.19	0.157	1.27 × 10^−13^
2	NA	Chr2 g.111588505GA>G	3.19	0.157	1.40 × 10^−13^
3	rs379031581	Chr2 g.111587292A>G	3.19	0.157	1.40 × 10^−13^
4	rs385337886	Chr2 g.111573853A>G	3.19	0.157	1.40 × 10^−13^
5	rs468881264	Chr2 g.111615661G>A	3.19	0.157	1.40 × 10^−13^
6	NA	Chr2 g.111601410A>G	3.18	0.156	1.41 × 10^−13^
7	rs381689348	Chr2 g.111604662A>C	3.18	0.156	1.41 × 10^−13^
8	rs377769439	Chr2 g.111634835G>A	3.18	0.157	1.55 × 10^−13^
9	rs385963805	Chr2 g.111570788G>A	3.18	0.157	1.55 × 10^−13^
10	rs380782402	Chr2 g.111560710G>A	3.17	0.156	1.58 × 10^−13^

^a^Effect size is expressed as the percentage of white on the coat attributed to each additional ‘Q’ allele

### Analysis of the significant loci on each detected chromosome

#### Chromosome 22

**Fig. 2:**
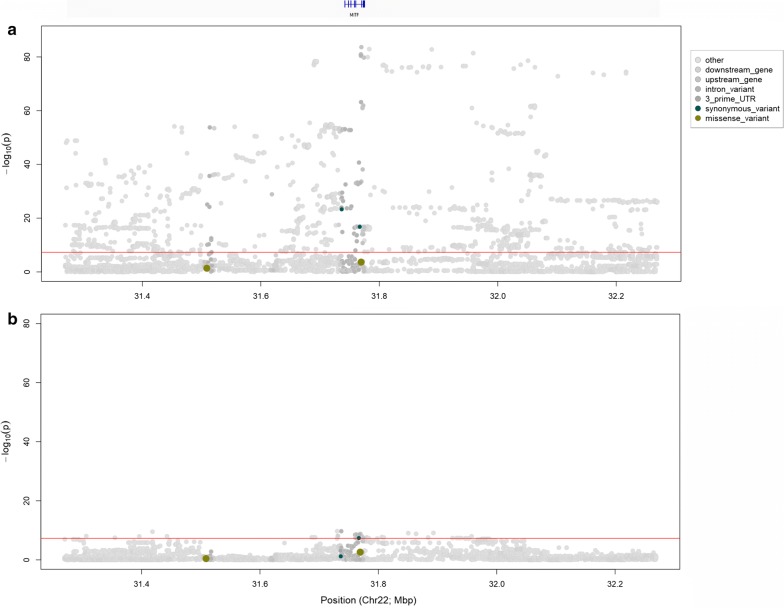
QTL analysis of chromosome 22 with variants color-coded according to predicted functional impact using SNPEff. **a** 1-Mb window of imputed whole-genome sequence association data centred around the top variant Chr22 g.31769747A>G (rs209784468) with the corresponding annotated gene track above. **b** 1-Mb window of imputed sequence association data with rs209784468 fitted as a fixed effect in the association model. The red line indicates the genome-wide significance threshold *p *= 5×10^−8^

A SNP at Chr22 g.31769747A>G (rs209784468) was identified as the most significant variant in our association analysis (*p* = 1.83 × 10^−79^), and mapped to a region 284-bp upstream of the Ensembl-annotated transcription start site (TSS) of the *MITF* gene. The MITF transcription factor is involved in melanocyte survival, maintenance and differentiation [29], and is therefore the most obvious candidate at this locus. Based on the Ensembl v92.31 gene build [25, 26], *MITF* is also one of the only two annotated protein-coding genes that are present within a 1-Mb window around rs209784468, which provides strong support for the causative status of this gene. Figure 2a shows a Manhattan plot of this interval, with the variants being color-coded according to predicted functional impact using SNPEff [24]. To assess whether the signal observed on chromosome 22 was likely representative of a single biallelic QTL, we ran an additional analysis, by fitting the top-associated SNP (rs209784468) as a fixed effect in the association model. This analysis removed significance at almost all the variants within a 1-Mb interval (Fig. 2a, b), but a slight residual signal remained (smallest *p* = 8.53 × 10^−11^ for Chr22 g.31730376 rs109549448; Fig. 2b). Although imputation error or unaddressed population stratification might explain the small residual signal revealed in this analysis, the well-described allelic heterogeneity for *MITF* supports the potential existence of multiple and/or multiallelic QTL. It should be noted that, in a recent analysis in Brown Swiss cattle, Hofstetter et al. [12] identified a SNP (rs722765315), located within the 5′-region of the *MITF* gene as a candidate causal variant for white spotting [12]. However, examination of this site in our whole-genome sequenced cohort shows that it is invariant in Holstein–Friesian and Jersey animals, which suggests the presence of one or more alternate causal variants in the New Zealand population.

*A novel, polymorphic MITF pseudogene as a candidate for the white spotting QTL* Notably, we observed a predicted missense mutation that affects *MITF* at Chr22 g.31769331C>T (rs110881545; Fig. 2a). Although it could be a candidate mutation for the QTL, this variant was not significant, and was called at a very low frequency in the genome sequence reference population used for imputation (MAF < 0.01). Manual inspection of sequence alignments from animals heterozygous for this variant showed read depth anomalies around annotated intron–exon boundaries, which led us to analyze in more detail these features. Although we used DNA-based sequence data, at these boundaries we observed an increased sequencing depth for the exons, which are reminiscent of RNA-sequence alignments (see Additional file 2: Figure S1). Analysis of soft-clipped reads from the exons showed that the mismatches corresponded to neighboring exon structures, which suggest that they were derived from a mis-mapped, processed *MITF* pseudogene. Non-exonic read pairs from the apparent *MITF* pseudogene mapped to a single location on chromosome 12 at 58.7-Mb, indicating that this locus is the likely site of integration of the pseudogene. Notably, this pseudogene was polymorphic across animals, which raised the possibility that the QTL might be caused by this structural variant. String match searching for spliced *MITF* sequence reads from the whole-genome sequence alignments, allowed us to genotype the 565 whole-genome-sequenced animals in our reference population for the pseudogene, giving a MAF of 0.026 for the integrated allele. This MAF value contrasted markedly with that of the top tag variant from GWAS (MAF = 0.304); and when pairwise linkage disequilibrium statistics were examined between the pseudogene ‘genotype’ and variants from the broader chromosome 22 and chromosome 12 regions, the most highly correlated markers were also non-significant in the GWAS (chromosome 12, maximum R^2^ = 0.72 for rs461882713 Chr12 g.6060748C>G, *p* = 0.72; chromosome 22, maximum R^2^ = 0.69 for rs384283283 Chr22 g.31734120C>T, *p* = 0.67). Although the processed *MITF* pseudogene was a good biological candidate for the modulation of coat color or pattern, these observations led us to assume that it was not responsible for the white spotting QTL in our study.

**Fig. 3:**
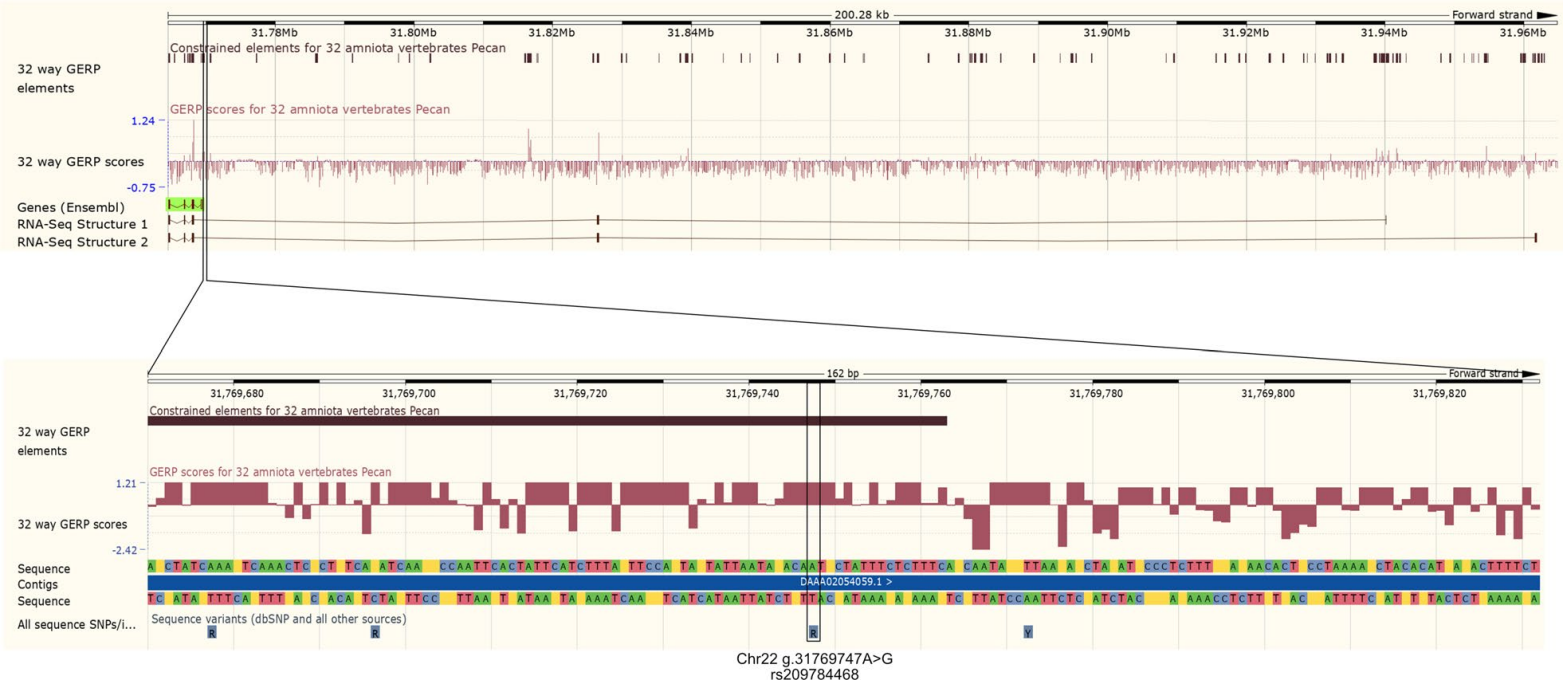
Detailed view of introns 1 to 3 of the Ensembl-derived *MITF* gene structure and introns 1 to 5 of the RNA-seq derived *MITF* structures, with constrained elements and GERP score for 32 amniota vertebrates from Ensembl (Bos taurus v92.31). g.31769747A>G (rs209784468) is highlighted and located to a highly conserved region within intron 2 of the RNA-seq derived MITF gene structures

*Evolutionarily conserved, candidate causative regulatory variants at the MITF locus* Apart from the *MITF* pseudogene identified above, no other protein-coding changes were identified in *MITF* that could explain this QTL. Although two synonymous *MITF* variants exceeded genome-wide significance, their association was sufficiently weak to discard them as underpinning the QTL (Fig. 2a). Together, these observations suggested an expression-based mechanism for a MITF-derived effect on white spotting. The top associated variant Chr22 g.31769747A>G (rs209784468) is a reasonable candidate in this regard, as it maps to a region immediately upstream of the annotated transcription start site (TSS). However, inspection of RNA-sequence (RNA-seq) data for black and white bovine skin samples published by Koufariotis et al. [22] showed alternative gene structures that include additional 5′ exons to the Ensembl-derived annotation (MITF-201; Ensembl v92.31), in which the rs209784468 variant mapped to intron 2 of the two predominant RNA-seq derived structures (Fig. 3). Similarly, examination of the transcripts annotated on the newest version of the bovine reference assembly at the time of the preparation of this paper (ARS-UCD1.2) showed alternative *MITF* structures, for which the skin-derived transcripts were best represented by the MITF-205 and MITF-206 transcripts (Ensembl v96.12). Notably, 18 additional variants that displayed association statistics that were broadly similar to those of rs209784468 (*p* < 5 × 10^−70^) also mapped within introns 1, 2, 3, and up to 100-kb upstream of the alternate *MITF* isoforms. To further investigate these variants, we downloaded genome evolutionary rate profiling (GERP) scores from the Ensembl portal to assess conservation metrics of the sites (Table 2; [25, 26]). Although the location of this variant was less appealing than some of the others that map closer to the assumed 5′ *MITF* promoter, the top-associated SNP is the only variant that mapped to a conserved element identified from the 32-way amniote vertebrate alignments (Fig. 3). This SNP is also conserved on a site-wise basis (GERP score = 1.21), and based on its association ranking, it constitutes a plausible candidate regulatory mutation for this QTL.

**Table 2:** Top variants mapping within introns 1, 2, 3 and up to 100-kb upstream of the annotated MITF TSS, with conservation (GERP) score for 32 amniota vertebrates (Ensembl Bos taurus v92.31—UMD3.1)

Variant reference ID	Genomic position	GERP score	Constrained element	p-value
rs209784468	Chr22 g.31769747A>G	1.21	Yes	1.83 × 10^−79^
rs461193589	Chr22 g.31783093T>C	0.07	No	8.67 × 10^−79^
NA	Chr22 g.31768931A>T	− 1.85	No	6.11 × 10^−77^
rs433645096	Chr22 g.31768933A>T	0.92	No	1.59 × 10^−76^
rs208958980	Chr22 g.31769772T>C	1.21	No	1.59 × 10^−76^
NA	Chr22 g.31768928TG>T	− 0.22	No	2.29 × 10^−76^
rs385179918	Chr22 g.31780393C>A	0	No	6.69 × 10^−76^
rs110372927	Chr22 g.31774043C>T	− 1.52	No	7.97 × 10^−76^
rs384965533	Chr22 g.31807384A>G	0.2	No	8.11 × 10^−73^
rs109143893	Chr22 g.31805754C>T	− 1.25	No	1.28 × 10^−72^
rs385825679	Chr22 g.31811182C>T	0	No	2.82 × 10^−72^
rs209226877	Chr22 g.31873774A>C	0.65	No	4.06 × 10^−72^
rs109756444	Chr22 g.31853470A>G	− 1.69	No	5.25 × 10^−72^
rs378395938	Chr22 g.31838217G>A	− 0.08	No	6.63 × 10^−72^
rs110467669	Chr22 g.31849617A>G	− 1.69	No	6.63 × 10^−72^
rs110989002	Chr22 g.31812468A>T	− 0.09	No	8.75 × 10^−71^
rs110743578	Chr22 g.31821264C>G	− 1.69	No	1.54 × 10^−70^
rs110276495	Chr22 g.31863698C>T	− 0.4	No	2.59 × 10^−70^

#### Chromosome 6

The top variant at the chromosome 6 locus (Chr6 g.64210286A>G rs451683615, *p* = 1.1 × 10^−64^), maps to an intergenic region approximately 280-kb downstream of the *KCTD8* gene, which represents quite a considerable distance from the *KIT* gene (~ 7.5-Mb). However, the third and fourth most strongly associated variants map within the fourth intron of *KIT* (Chr6 g.71873479T>C rs109512689, *p* = 8.08 × 10^−61^ and Chr6 g.71873455A>C rs385773341, *p* = 8.08 × 10^−61^).

**Fig. 4:**
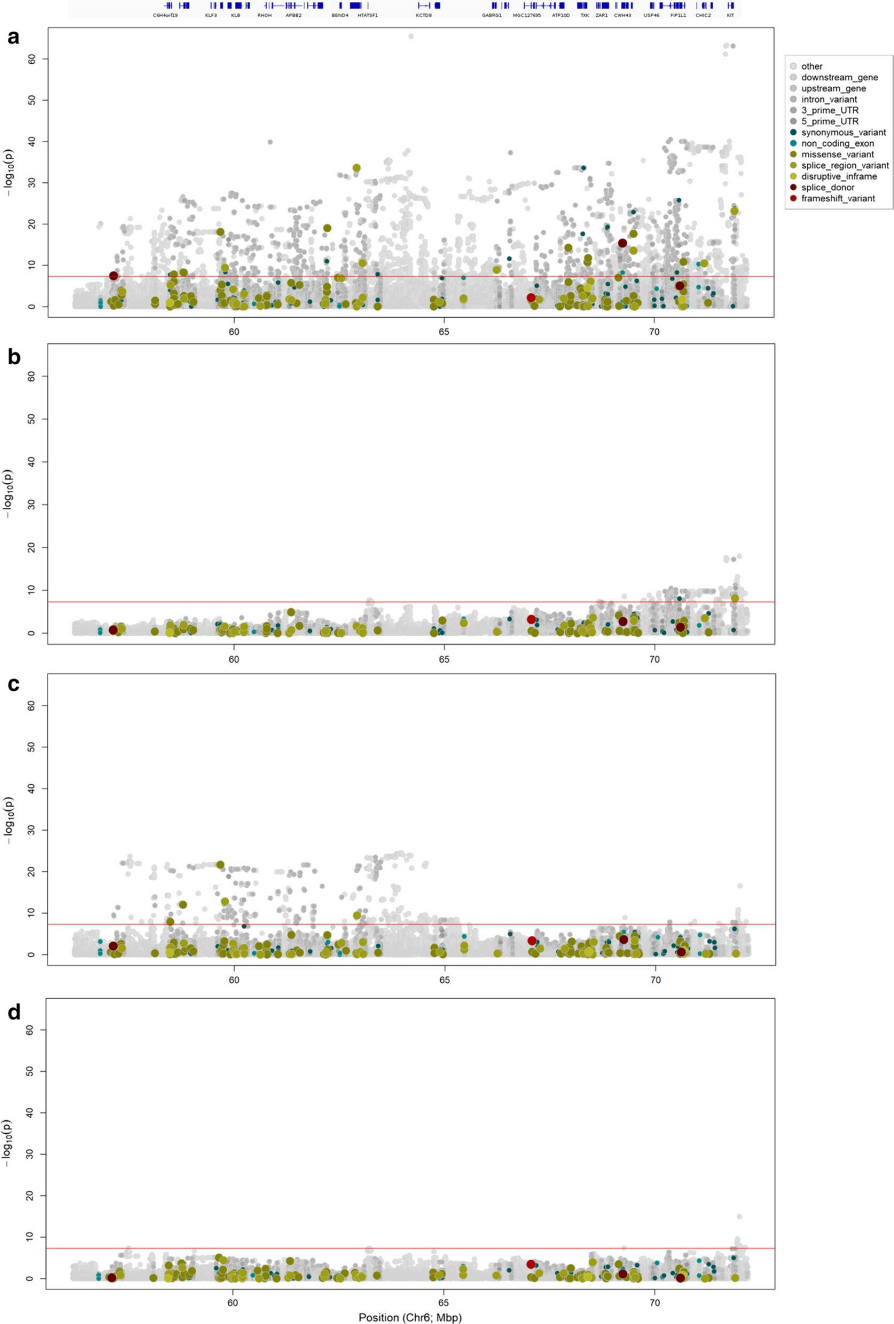
QTL analysis of chromosome 6 with variants color-coded according to predicted functional impact using SNPEff. **a** 16-Mb window of imputed whole-genome sequence association data centred around the top variant Chr6 g.64210286A>G (rs451683615) with the corresponding annotated gene track above. **b** 16-Mb window of imputed whole-genome sequence association data with rs451683615, **c** Chr6 g.71722665C>T (rs463810013) and **d** both rs451683615 and rs463810013 fitted as fixed effects. The red line indicates the genome-wide significance threshold *p* = 5 × 10^−8^

Given the dispersion of the chromosome 6 signal, and the association of variants that are located within and adjacent to the strong a priori candidate gene *KIT*, we considered a large interval (16-Mb) around the top variant rs451683615 for functional prediction of variant effects. The following genes map to this interval: *C6H4orf19, TBC1D1, KLF3, TMEM156, KLB, UBE2K, RHOH, RBM47, APBB2, UCHL1, BEND4, SHISA3, HTATSF1, KCTD8, YIPF7, GABRG1, GABRA2, MGC127695, GABRB1, ATPq0D, NFXL1, TXK, SLAIN2, OCIAD1, LRRC66, USP46, SCFD2, FIP1L1, UFM1, GSX2, KIT, KDR, SRD5A3, PDCL2* and *CREP135*. Figure 4 shows a Manhattan plot for this region, with variants color-coded according to predicted functional impact using SNPEff. Based on association statistics, none of the variants in the top 10 orders of magnitude are predicted to change the protein-coding sequence of these genes, although there is a modestly associated splice region variant in *KIT* (Chr6 g.71906518T>C rs109750754, *p* = 1.94 × 10^−23^). Given that the primary signals highlight non-coding variants, a QTL mechanism that incorporates one or more gene expression-based effects seems most likely.

*Multiple segregating QTL at the KIT locus* One explanation for the dispersed nature of the chromosome 6 QTL is that this locus comprises multiple, overlapping effects. Linkage disequilibrium (LD) analysis between the top variant (Chr6 g.64210286A>G rs451683615) and the next three most strongly associated variants (Chr6 g.71722665C>T rs463810013, Chr6 g.71873479T>C rs109512689 and Chr6 g.71873455A>C rs385773341) supports this hypothesis, with rs451683615 being in relatively low LD with the other variants (maximum R^2^ = 0.35). Furthermore, when rs451683615 was fitted as a fixed effect, the signal on chromosome 6 still exceeded the genome-wide significance threshold (*p* = 5 × 10^−8^), with the two strongly correlated *KIT* variants (R^2^ = 0.91) rs208251862 (Chr6 g.71692344C>A; *p* = 7.1 × 10^−19^) and rs463810013 (*p* = 1.5 × 10^−18^) now being the top variants (Fig. 4b). When the rs463810013 variant was fitted as a fixed effect to represent these effects, rs451683615 once again became the most significant variant (*p *= 3.054 × 10^−25^; Fig. 4c), and when both rs451683615 and rs463810013 were fitted as fixed effects, a small signal was still detected near *KIT* (smallest *p* = 3.31 × 10^−11^ for Chr6 g.72007252A>T rs109258078; Fig. 4d). These results suggest that the signal observed on chromosome 6 is likely the result of two or more QTL, and/or alternatively, the consequence of one or more structural variants that are not well tagged, and therefore cannot be easily accounted for by fitting biallelic SNPs in the association models.

*Structural variant analysis at the chromosome 6 locus.* Given the ambiguity of the association signals at the chromosome 6 locus, and the implication of *KIT* structural variants that have a role in other coat characters in cattle (e.g. white face piebaldism in Hereford [30] and color-sidedness in Belgian Blue, Brown Swiss, and other breeds [31]), we performed a sequence-based structural variant analysis to attempt to identify segregating candidate mutations for this QTL. This analysis was conducted using the same population of 565 whole-genome-sequenced animals as that used for sequence imputation prior to GWAS, and we focused on a broad 20-Mb region (60 to 80-Mb) to capture the dispersed nature of the association peak. This region included the top 10 variants shown in Table 1, for which the CNVnator software (v0.3.3) [28] was used to call structural variants within the interval based on a 1-kb sliding window approach with 500-bp overlaps (see Methods). This analysis revealed a large number of candidate polymorphic intervals (N = 39,960). We used correlation analysis between estimated copy numbers and genotypes from the top two associated GWAS variants (Chr6 g.64210286A>G rs451683615 and Chr6 g.71722665C>T rs463810013) to prioritize the variants for subsequent investigations. Of the top 10 most highly correlated variants for each of the two tag SNPs, these intervals represented six discrete structural variants (and some variants could be merged because they spanned adjacent intervals). Table 3 shows the position, mutation-type, and LD correlation coefficient of these six variants for the tag-SNP of interest, with LD values based on re-calling of the intervals following merging and manual boundary refinement. Visualization of sequence alignments suggested legitimate polymorphic structural variation for all six variants, with four of these showing clear multi-modality in read depth (see Additional file 2: Figure S2). Notably, LD analysis between the six structural variants and the 124,445 other sequence variants within the 20-Mb chromosome 6 interval showed that five of the six variants were better tagged by other chromosome 6 polymorphisms, which all showed limited phenotypic association by comparison with the top-associated tag SNPs rs451683615 and rs463810013 (Table 3). One exception was a 330-bp duplication at Chr6:72,060,120-72,060,450 bp, where this variant was best tagged by a SNP that is largely equivalent to rs463810013 (rs385773341 Chr6 g.71873455A>C; R^2^ = 0.98 with rs463810013; Table 3). None of the six structural candidates mapped to protein coding sequences, although the apparent 330-bp duplication was also the polymorphism nearest to *KIT* (albeit 142-kb downstream). Assessment of the potential function for this variant did not present any obvious regulatory implication, since the duplication was devoid of noteworthy site-wise conservation or GERP-annotated constrained elements. Acknowledging the fact that our read-depth-based analysis of structural variation may represent the complexity of the identified candidate mutations, these data likely exclude five of six of the structural variants as candidates for the white spotting QTL. The potential role of the sixth candidate variant is unknown, and although the duplication was best represented by the top GWAS tag variants, its overall correlation was still low (maximum R^2^ = 0.43). This observation, and the fact that copy number genotypes were not clearly differentiated for this variant (see Additional file 2: Figure S2), lead us to suggest that physical genotyping and more detailed investigation will be required to further assess the nature and candidacy of this polymorphism.

**Table 3:** Description and LD summary statistics for the candidate structural variants that are most highly correlated with tag SNPs rs451683615 (Chr6 g.64210286A>G) and rs463810013 (Chr6 g.71722665C>T)

Region spanning CNV	Type	rs451683615 correlation (R^2^)	rs46381013 correlation (R^2^)	Closest gene	Maximum R^2^	SNP ID	GWAS p-value
Chr6:64,092,201–64,092,752 bp	Deletion	0.172	0.099	KCTD8	0.544	rs110545184	3.24 × 10^−22^
Chr6:65,557,508–65,559,004 bp	Deletion	0.102	0.066	GNPDA2	0.876	rs384078363	3.74 × 10^−5^
Chr6:65,657,051–65,657,595 bp	Deletion	0.128	0.089	GNPDA3	0.746	rs383024906	2.79 × 10^−11^
Chr6:68,269,498–68,270,804 bp	Deletion	0.171	0.164	NFXL1	0.569	rs456305543	5.89 × 10^−34^
Chr6:71,310,834–71,312,202 bp	Deletion	0.065	0.163	GSX2	0.695	rs466525306	4.78 × 10^−12^
Chr6:72,060,120–72,060,450 bp	Duplication	0.22	0.431	KIT	0.432	rs385773341	8.08 × 10^−61^

*CNV* copy number variant, *R*^*2*^ linkage disequilibrium correlation coefficient, *SNP ID* single nucleotide polymorphism accession number

#### Chromosome 2

**Fig. 5:**
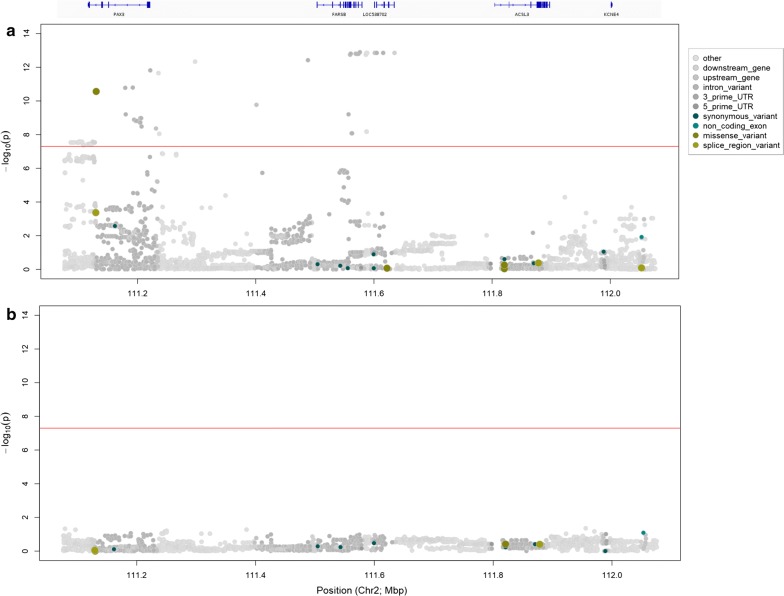
QTL analysis of chromosome 2 with variants color-coded according to predicted functional impact using SNPEff. **a** 1-Mb window of imputed whole genome sequence association data centred around top variant Chr2 g.111576221A*>*C (rs109979909) with the corresponding annotated gene track above. **b** 1-Mb window of imputed whole-genome sequence association data with rs109979909 fitted as a fixed effect. The red line indicates the genome-wide significance threshold *p* = 5 × 10^−8^

The top variant at the chromosome 2 locus (Chr2 g.111576221A>C rs109979909, *p* = 1.27 × 10^−13^) maps to intron 1 of the *FARSB* gene. Considering all the genes in a 1-Mb interval centered on rs109979909, the *PAX3, MIR2284Y*-*5, FARSB, LOC538702, MOGAT1, ACSL3, RPSL3, RPS6* and *KCNEE4* genes map to this region. In particular, *PAX3* is a striking candidate, since it encodes a *MITF* transcription factor (see “Chromosome 22” section above) and was proposed as a causal gene for the ‘splashed white’ coat phenotype in horses [4]. Variant effect prediction for all variants in the 1-Mb interval (Chr2:111,076,221–112,076,221 bp) revealed a candidate causal missense mutation in *PAX3*, that codes for a threonine to methionine substitution at amino acid position 424 (rs208582518; p.Thr424Met; Fig. 5; [32]). Although the p.Thr424Met variant shows a comparatively weaker association than the top associated variant at this locus (*p* = 2.72 × 10^−11^ versus smallest *p* = 1.27 × 10^−13^), it is sufficiently strongly associated to remain a compelling candidate mutation for the QTL. Additional inspection of the sequence alignments across the 1-Mb region centered on rs109979909 did not show any evidence of segregating structural variants as alternative candidates at this locus.

**Fig. 6:**
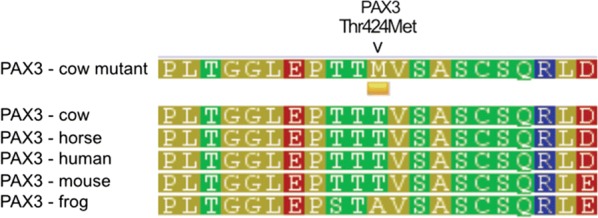
Region around the p.Thr424Met mutation. Wild-type threonine at position 424 is conserved across cow (*Bos taurus*), horse (*Equus caballus*), human (*Homo sapiens*) and mouse (*Mus musculus*) *PAX3* orthologues

*A novel candidate causal PAX3 missense mutation* The p.Thr424Met (rs208582518) variant maps to exon 9 of the *PAX3* gene. When fitted as a fixed effect in the association model, the variant accounted for the majority of the signal at this locus (smallest *p* = 0.0149 for Chr2 g.111955758G>A rs41718011 for this model; Fig. 5b). The p.Thr424Met variant is located within the transactivating domain of the PAX3 transcription factor, which is also identified as a constrained element from the GERP 32-way amniote alignments. The variant has a site-wise GERP score of 1.72, and when assessing the predicted functional impact of the missense variant using the SIFT algorithm [33] that is integrated as part of the Ensembl Variant Effect Predictor [32], this SNP is predicted to be ‘deleterious’ (score 0.01, low confidence). Likewise, p.Thr424Met is predicted to be ‘possibly damaging’ (score 0.86) by the Polyphen-2 functional prediction tool [34, 35], and multiple alignment of PAX3 protein sequences representing a range of vertebrates also shows conservation of the threonine residue and surrounding amino acid acids in mammals (Fig. 6). Overall, the *PAX3* p.Thr424Met missense mutation is a compelling candidate causal mutation for the white spotting phenotype, although the strong association of other non-coding variants leaves open the possibility of expression-based effects, which again operate most likely through the *PAX3* gene.

### Breed, frequency, and effect size characteristics of the three major QTL

White spotting is a characteristic trait in HF and has been under selection for many generations. Although some J animals in New Zealand show white spotting, it is far less frequent in this breed. Thus, we expect that the alleles that are associated with a greater proportion of white spotting are more frequent in HF. Based on the allele frequencies of the top tag variants for each of the three major QTL in 589 purebred HF, and 274 purebreed J, we obtained the frequencies shown in Table 4 (see “Methods”: Study populations for breed definitions). Here, we have denoted the white-increasing allele as *Q*, and the white decreasing allele as *q*.

**Table 4:** *Q* allele frequencies for the top variant at each QTL for 589 purebred Holstein*–*Friesians and 274 purebred Jerseys

Genomic position	Variant reference ID	*q* allele	*Q* allele	HF *Q* frequency	J *Q* frequency
Chr22 g.31769747A>G	rs209784468	G	A	0.97538	0.3431
Chr6 g.64210286A>G	rs451683615	G	A	0.99236	0.6332
Chr2 g.111576221A>C	rs109979909	C	A	0.98557	0.6953

**Fig. 7:**
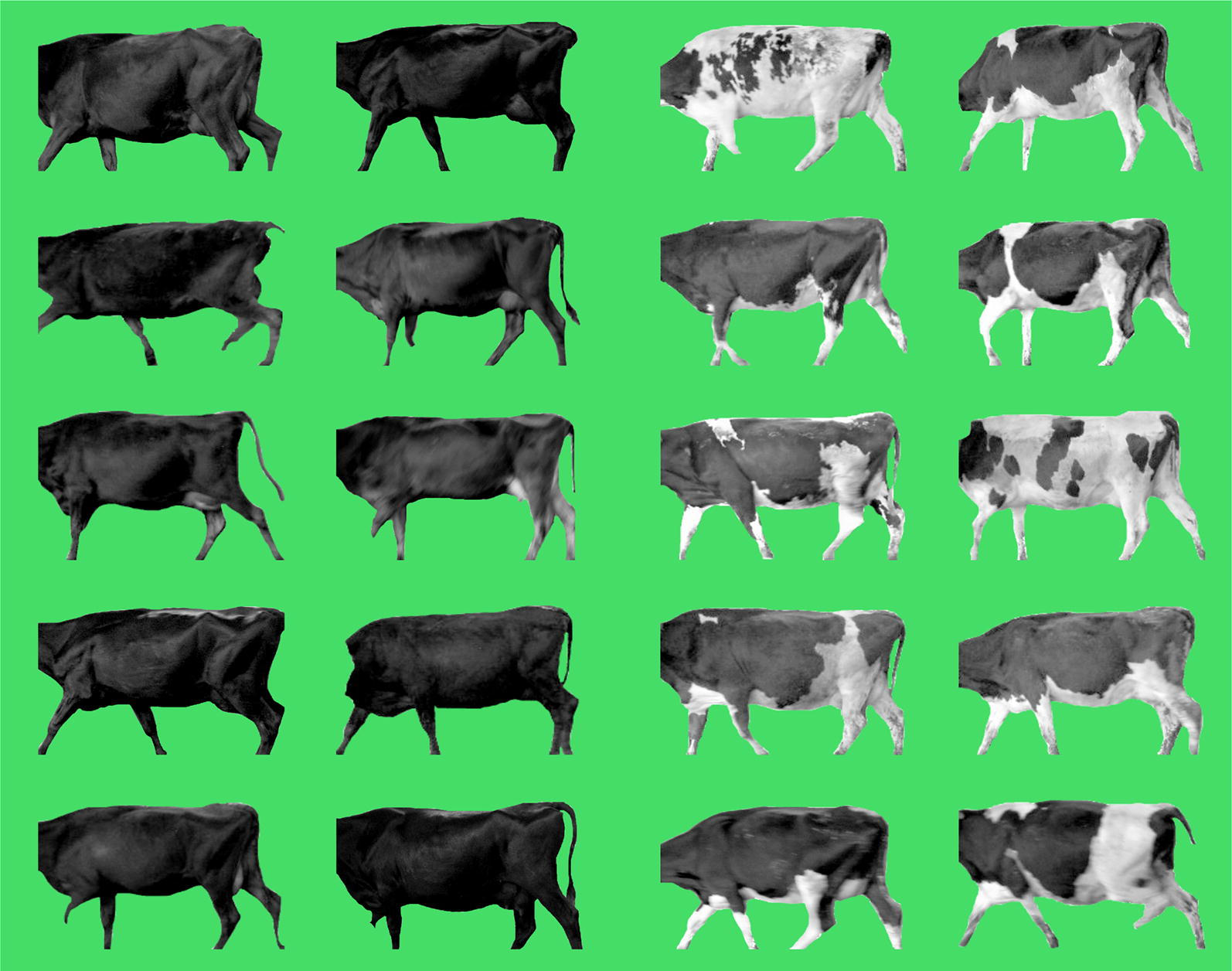
Black and white images of 10 ½HF × ½J cows carrying the smallest number of *Q* alleles observed (2*Q*; left), contrasted with 10 ½HF × ½J cows carrying the [maximum number of *Q* alleles at the three major loci (6*Q*; right)

Given the large sizes of the effect of the QTL, it is interesting to examine how ‘*Q*’ (more white) or conversely ‘*q*’ (less white) alleles might combine across loci to impact the phenotype. To investigate the QTL in this way, ‘stacked’ genotypes were derived for each animal based on the top-associated tag variants representing the chromosome 2, 6, and 22 loci. In this way, animals could be categorized based on the number of ‘*Q*’ alleles presented (possible range from 0 to 6). This analysis focused on a subset of 699 F2 cows (½HF × ½J) to minimize possible confounding by admixture, where animals were also all derived from the same research group. The smallest number of *Q* alleles carried in this population was two (‘2*Q*’; N = 10 cows), none of these cows displaying visible white color on their coat (based on pictures that show only a single side view). By comparison, animals that carry six *Q* alleles (i.e. homozygous *Q* for all three loci; ‘6*Q*’; N = 160) displayed a striking increase in white spotting. Figure 7 compares the 10 2*Q* animals (left panel) with a random selection of 10 6*Q* animals (right panel), and highlights the major impact of these QTL. The mean percentage of white spotting value was 0% for the 10 2*Q* animals and 32.6% for the 160 6*Q* animals (or 36.9% for the subset of 10 6*Q* animals shown in Fig. 7). These observations give some clue as to the somewhat counterintuitive finding that *Q* alleles for two of the three QTL are the major alleles in J animals. Although this breed is best known for its solid, light brown coat, in F2 animals, only those with a large number of *Q* alleles showed substantial proportions of white spotting on their coat. Additional file 2: Figure S3 and Additional file 1: Table S2 also show a breakdown of the *Q* allele counts in purebreds, based on the 589 HF and 274 J animals referenced above. In this purebred dataset, the percentage of J animals with 6*Q* alleles is only 1.8%, whereas in HF it reaches 91.7%. This is consistent with the observation that the numbers of J animals in New Zealand with prominent white spotting are small and the numbers of those that have splashes of white or white accents are larger. It is also noteworthy that the *Q* alleles for the three major QTL are reference alleles in the UMD3.1 genome assembly, which is based on a single Hereford cow. The population frequencies of these variants in the Hereford breed are unknown, and although this breed is not as characteristically spotted as the Holstein breed, Herefords are well known for their white faces (attributed to another mutation in *KIT* [30]), with substantial white markings concentrated on the belly, brisket, neck, and back.

## Discussion

We present the first association analysis for white spotting in dairy cattle using imputed whole-genome sequence data. This study comprises the largest GWAS for this phenotype, to date, providing details of the genetic effects on white spotting in a population of approximately 3000 HF, J, and their crosses. We provide evidence for the implication of the *KIT*, *MITF* and *PAX3* genes in white spotting of the coat, and further suggest regulatory and missense variants that potentially explain the effects of the *MITF* and *PAX3* genes.

*MITF* is the only plausible candidate for the QTL on chromosome 22, which encodes a transcription factor that has been shown to impact pigmentation in cattle [12, 36], mice [37], horses [4, 5], dogs [38, 39], humans [40], and most recently ducks [41]. It is also the only gene located near the top associated variant (Chr22 g.31769747A>G rs209784468), which is situated in intron 2 of MITF transcripts based on the analysis of skin RNA-seq data. The rs209784468 variant falls within a conserved genomic region, which, in conjunction with its status as the lead associated variant, makes rs209784468 a candidate causal variant for this QTL. Given that this SNP and other lead variants are non-coding, and given the lack of other candidate variants that map to protein-coding sequences, we hypothesize that the mechanism underlying the QTL on chromosome 22 is a modulation of the expression of *MITF*. However, how this effect manifests itself during development is unknown. MITF is required during embryonic development to stimulate the transition of neural crest cells into melanocyte precursors [42]. If the *MITF* gene is not expressed within the small window during which transition is meant to take place, future expression of *MITF* cannot rescue melanocyte development [42]. Impaired functionality or expression of the *MITF* gene during development will result in a reduced number of melanocytes, and manifest itself as white spotting on the coat [42]. However, impaired functionality of the *MITF* gene within the mature hair follicle may also impair melanocyte survival and differentiation [29], thus decreasing the number of pigment producing melanocytes. In humans and mice, loss-of-function mutations in *MITF* cause severe symptoms including: coloboma, osteopetrosis, microphthalmia, albinism and deafness [43, 44]. Disruptive mutations in *MITF* also cause Tietz syndrome, which is characterized by depigmentation of the skin, hair, iris and severe hearing loss, and Waardenburg syndrome type 2A, which is characterized by patchy depigmentation of the skin and bi- or unilateral deafness in humans and mice [37, 40, 45]. Interestingly, mutations with a strong effect have also been observed in cattle [36, 46]. The white spotting *MITF* variant that we describe in this study represents a common allele (or nearly fixed in the case of HF animals), with no known effects on hearing or other undesirable phenotypes. The fact that this variant causes a less severe phenotype than the variants with a strong effect fits with an expression-based mechanism for this QTL, however it would still be interesting to compare the phenotype of the segregating individuals for the QTL identified in the current analysis with the phenotypes of individuals with more severe *MITF* syndromes (e.g. hearing loss). In terms of functional analyses, to unambiguously test the role of the rs209784468 SNP and other linked candidates, experiments analogous to those performed in an investigation of human hair color loci [47] could be performed. Cell-culture-based analyses or studies on model organisms could be conducted to perturb the candidate loci that have an effect on gene expression or pigment formation/melanocyte function.

The most significant variant for the QTL on chromosome 6 mapped to a region 7.5-Mb upstream of the *KIT* gene. Although seemingly too far away to cause this signal, the *KIT* gene is perhaps the single most famous and well-characterized pigmentation gene. There are 19 reported mutations within or near the equine *KIT* gene that cause either complete depigmentation, or white spotting [3, 5, 6, 48], and there are approximately 76 known *KIT* alleles in mice that cause dominant or semi-dominant white spotting [9, 49]. A *KIT* translocation mutation has also been identified as the causative mutation for ‘color sidedness’ and the white coat phenotype in Belgian Blue and White Galloway cattle [31, 50]. Although it is possible that the white spotting QTL in the current study is underpinned by contributions from other genes, these facts make *KIT* worthy of consideration as the likely causal agent underlying the chromosome 6 signals. Thus, the inconsistency of the mapping data may instead represent an amalgamation of multiple signals at the locus, and/or some other complexity that is not well represented by our imputed genome sequence dataset. Indeed, when the lead variants were consecutively fitted in our association analyses, no single variant could account for the signal. Given the precedent regarding the *KIT* structural mutations that influence coat phenotypes, we also conducted a sequence-based structural analysis of a broad, 20-Mbp region encompassing *KIT* and the top tag variants from the GWAS. This analysis did not reveal any obvious candidate but it is possible that these efforts were confounded by errors in the genome assembly around *KIT*, an observation highlighted through analyses by Whitacre et al. [30]. If such confounders exist, breed-specific de novo assemblies and sequence information based on long-read sequencing technologies, such as single-molecule sequencing [51], may be helpful in future investigations of the locus. Additional future work could also attempt to fine map the effects in alternative breeds in which fewer QTL could be segregating, or alternatively conduct functional analyses as mentioned in the previous section for the associated variants that map to intron 4 of *KIT* itself.

To our knowledge, the observation of a likely role for *PAX3* in white spotting of the coat in cattle is a novel finding. The top variant for this QTL on chromosome 2 mapped to a region 0.3-Mb upstream of the *PAX3* gene, although bioinformatic prediction of variant effects revealed a highly associated p.Thr424Met missense mutation that could underlie this QTL. Previous studies have reported variants in *PAX3* that cause pigmentation phenotypes in humans [52], mice [53] and horses [4, 5] and variation in ambilateral circumocular pigmentation in the Fleckvieh breed of cattle [54]. The latter phenotype describes pigmentation of the area that encircles the animals’ eyes in breeds that otherwise have a white head, which raises the possibility that white spotting in HF is influenced by the same QTL that is involved in ambilateral circumocular pigmentation in Flekvieh cattle. In humans, as for some mutations in *MITF,* protein-changing variants in *PAX3* have been shown to cause a similar form of Waardenburg syndrome, which is characterized by wide set eyes, hearing loss and regions of depigmentation in the iris, hair and skin [52, 55]. Studies in humans and mice have demonstrated that the *PAX3* gene encodes a transcription factor that binds directly to the proximal M promoter of the *MITF* gene, thus facilitating expression of *MITF* [29, 55–57]. Studies of different spontaneous and radiation-induced *PAX3* mutations in Splotch mice have suggested that *PAX3* is required for proper development of neural crest cells, expansion of melanoblast populations, and prevention of melanoblast terminal differentiation [53]. Thus, if the function of the PAX3 protein is altered, *MITF* transcription and activity may be impaired, which in turn may have an impact on regional melanocyte populations and melanogenesis, resulting in an increased proportion of white spotting on the animal’s coat. It is also interesting that Hayes et al. [1] observed an association between variants that are located next to the bovine *PAX5* gene and the proportion of black on the coat. We did not observe a genome-wide significant signal on chromosome 8, although this association was demonstrated in Australian Holsteins [1]; the highlighted tag SNP in their study was not tested for association here because it was nearly fixed in our population (MAF < 0.001) and was excluded from the dataset. Unlike *PAX3*, the associations of *PAX5* and *MITF* with melanogenesis are unclear, but the implication of these two structurally related transcription factors in independent GWAS should be analyzed in future work. Regarding the other major QTL identified, functional studies are required to confirm a causative effect of the *PAX3* p.Thr424Met mutation, and confirm the molecular mechanism through which this QTL acts.

## Conclusions

Our results add strength to previous analyses that suggest the involvement of the *KIT* and *MITF* genes in white spotting of the coat in cattle, and reveal a new QTL for this trait at the *PAX3* locus. The genes identified highlight the commonality of the mechanisms that underlie the modulation of skin and hair pigmentation in animals, in which all three genes are key regulators of melanocyte development, migration, and differentiation. Moreover, these three genes have already been implicated in the modulation of pigment phenotypes in diverse species. In addition, the sizes of the effect of the major QTL being substantial, there is potential for selection of whiter or darker animals, depending on the farmers’ preferences.

## Supplementary information

**Additional file 1: Table S1.** Absolute number of animals genotyped per SNP Chip and number of SNPs per chip. Some cattle were genotyped on more than one panel, and thus they are included in multiple categories. The number of SNPs per panel presented in this table reflect number prior to filtering based on quality metrics. **Table S2.** Number of purebred Jerseys and Holstein–Friesians carrying 0-6*Q* alleles and corresponding mean percentage of white value. The mean percentage of white value reported is representative of raw phenotype measurements in purebred J and HF cattle from the mapping population. No fixed effects have been fitted to account for population structure or other confounding effects during this calculation.

**Additional file 2: Figure S1.** Read depth anomalies at intron–exon boundaries of *MITF* around exon 4 suggest the presence of a pseudogene. The top sequence alignment track represents a whole-genome sequenced animal heterozygous for the Chr22 g.31769331C>T (rs110881545) variant, for which read-depth is increased across the exons and soft-clipped reads show evidence of mismatches to neighbouring exon structures. **Figure S2.** Frequency of CNVnator assigned copy number across 565 sequenced cattle for each of the six candidate structural variants identified at the chromosome 6 locus. Four of the six structural variants show clear evidence of multimodality. **Figure S3.** Distribution of *Q* allele counts for each tag variant and combined across loci in cattle identified as purebred Holstein–Friesian (left) and pure-bred Jersey (right) within the population used for mapping.

## Data Availability

Phenotypic data representing the white spotting phenotype were uploaded as a submission to the Dryad database (10.5061/dryad.tqjq2bvtf) [58]. Sequence-based genotype data representing the three QTL of interest were uploaded under the same submission ID. Additional genome-wide data are available upon reasonable request following execution of a transfer agreement, and with permission of Livestock Improvement Corporation.
