# Supplementary material for: Genome-wide association analysis reveals QTL and candidate mutations involved in white spotting in cattle
Source: Genet Sel Evol. 2019 Nov 8;51:62. doi: 10.1186/s12711-019-0506-2 (PMC6839108; doi:10.1186/s12711-019-0506-2)
Supplement: Supplementary file 1 — Additional file 1: Table S1. Absolute number of animals genotyped per SNP Chip and number of SNPs per chip. Some cattle were genotyped on more than one panel, and thus they are included in multiple categories. The number of SNPs per panel presented in this table reflect number prior to filtering based on quality metrics. Table S2. Number of purebred Jerseys and Holstein–Friesians carrying 0-6Q alleles and corresponding mean percentage of white value. The mean percentage of white value reported is representative of raw phenotype measurements in purebred J and HF cattle from the mapping population. No fixed effects have been fitted to account for population structure or other confounding effects during this calculation. [file 12711_2019_506_MOESM1_ESM.docx]

**Table S1** Absolute number of animals genotyped per SNP Chip and number of SNPs per chip

| Genotyping Platform | | | | | | | | | | | | |
| --- | --- | --- | --- | --- | --- | --- | --- | --- | --- | --- | --- | --- |
|  | 50kv1 | 50kv2 | GGP50k | GGP50v1.1 | GGPHDv2 | GGPv1 | GGPv2 | GGPv2.1 | GGPv3 | GGPv3.1 | GGPv4 | HD |
| Animals | 600 | 1,297 | 1,051 | 109 | 156 | 334 | 202 | 6 | 726 | 100 | 180 | 458 |
| SNPs | 53,126 | 53,629 | 48,156 | 48,161 | 138,419 | 8,729 | 20,012 | 20,015 | 31,813 | 31,945 | 37,092 | 772,235 |

Some cattle were genotyped on more than one panel, so included in multiple categories. The number of SNPs per panel presented in this table reflect numbers prior to filtering based on quality metrics.

***Table S2*** *Number of purebred Jerseys and Holstein-Friesians carrying 0-6Q alleles and corresponding mean percentage of white value.*

|  | **Number of Q alleles** | | | | | | |
| --- | --- | --- | --- | --- | --- | --- | --- |
|  | **0** | **1** | **2** | **3** | **4** | **5** | **6** |
| **Jersey N** | 1 | 13 | 47 | 85 | 91 | 32 | 5 |
| **Jersey – mean percentage white** | 0 | 0 | 0.08 | 0.8 | 1.5 | 4.2 | 30.4 |
| **Holstein-Friesian N** | 0 | 0 | 0 | 1 | 4 | 44 | 540 |
| **Holstein-Friesian – mean percentage white** | - | - | - | 0 | 4.3 | 15.0 | 26.6 |

*The mean percentage of white value reported is representative of raw phenotype measurements in purebred J and HF cattle from the mapping population. No fixed effects have been fitted to account for population structure or other confounding effects during this calculation.*
