# Supplementary material for: Genome-wide association analysis reveals QTL and candidate mutations involved in white spotting in cattle
Source: Genet Sel Evol. 2019 Nov 8;51:62. doi: 10.1186/s12711-019-0506-2 (PMC6839108; doi:10.1186/s12711-019-0506-2)
Supplement: Supplementary file 2 — Additional file 2: Figure S1. Read depth anomalies at intron–exon boundaries of MITF around exon 4 suggest the presence of a pseudogene. The top sequence alignment track represents a whole-genome sequenced animal heterozygous for the Chr22 g.31769331C>T (rs110881545) variant, for which read-depth is increased across the exons and soft-clipped reads show evidence of mismatches to neighbouring exon structures. Figure S2. Frequency of CNVnator assigned copy number across 565 sequenced cattle for each of the six candidate structural variants identified at the chromosome 6 locus. Four of the six structural variants show clear evidence of multimodality. Figure S3. Distribution of Q allele counts for each tag variant and combined across loci in cattle identified as purebred Holstein–Friesian (left) and pure-bred Jersey (right) within the population used for mapping. [file 12711_2019_506_MOESM2_ESM.docx]

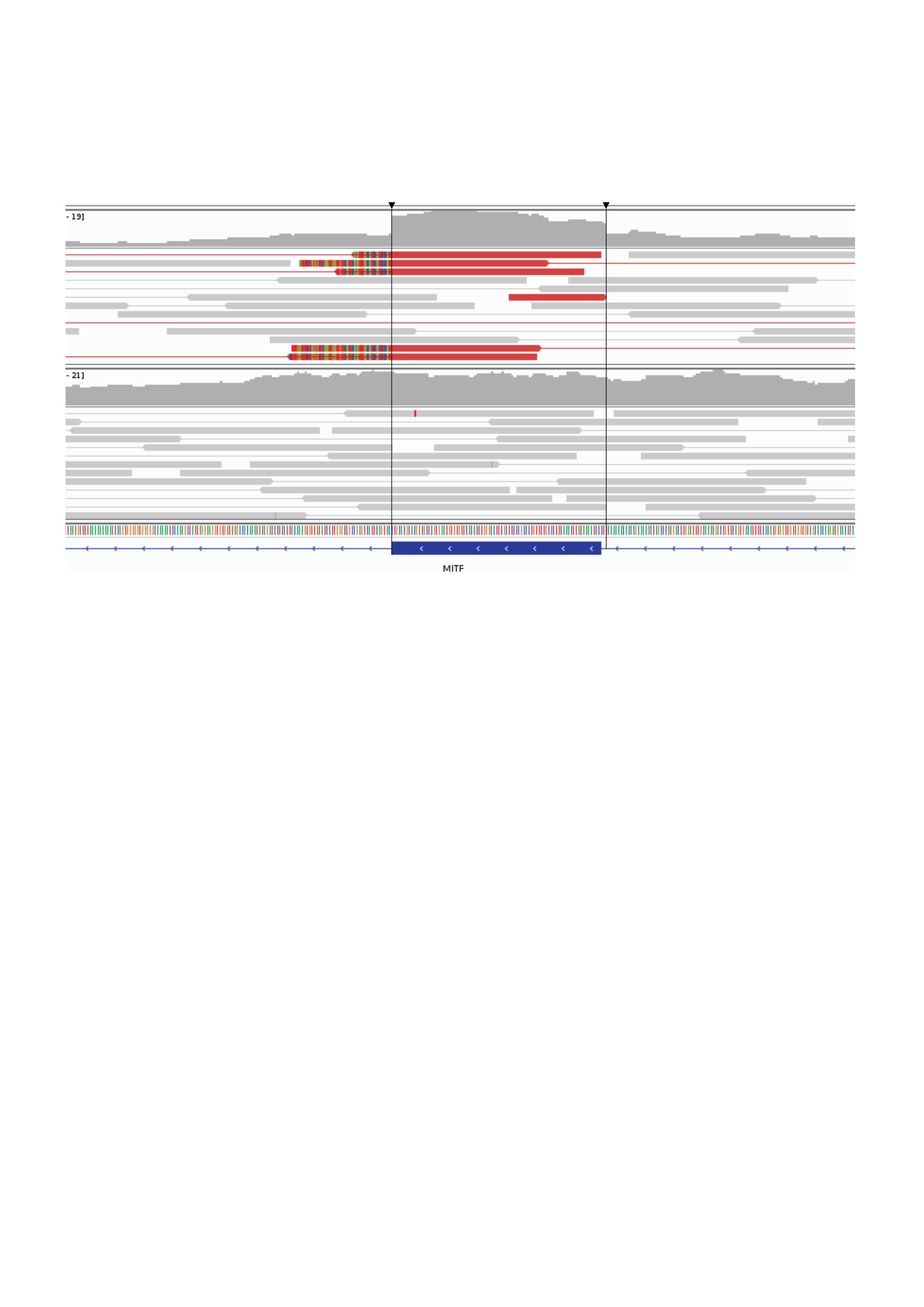


**Figure S1** Read depth anomalies at MITF intron-exon boundaries around exon 4 suggest the presence of a pseudogene. The top sequence alignment track represents a whole genome sequenced animal heterozygous for the Chr22 g.31769331C>T (rs110881545) variant, where there is increased read-depth across the exon and soft-clipped reads show evidence of mismatches to neighbouring exon structures.


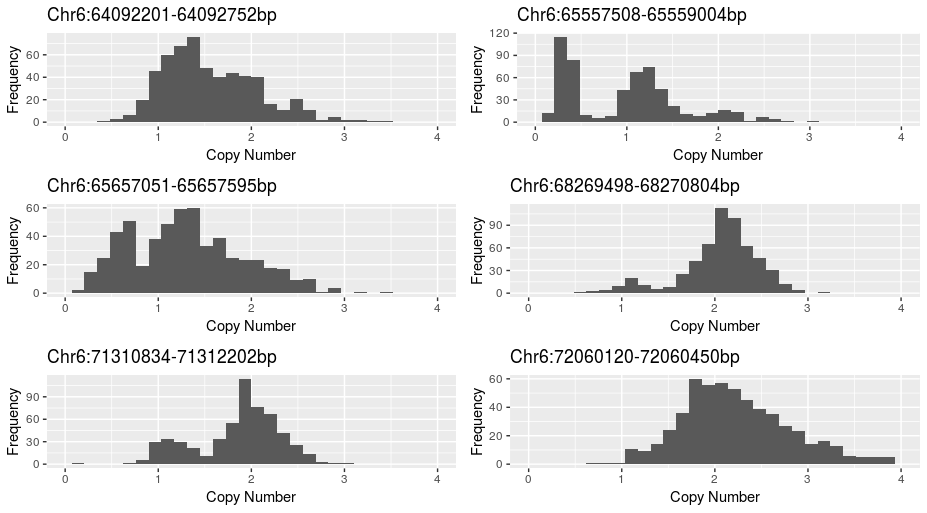


**Figure S2** Frequency of CNVnator assigned copy number across 565 sequenced cattle for each of the six candidate structural variants identified at the chromosome 6 locus. Four of the six structural variants here show clear evidence of multimodality.


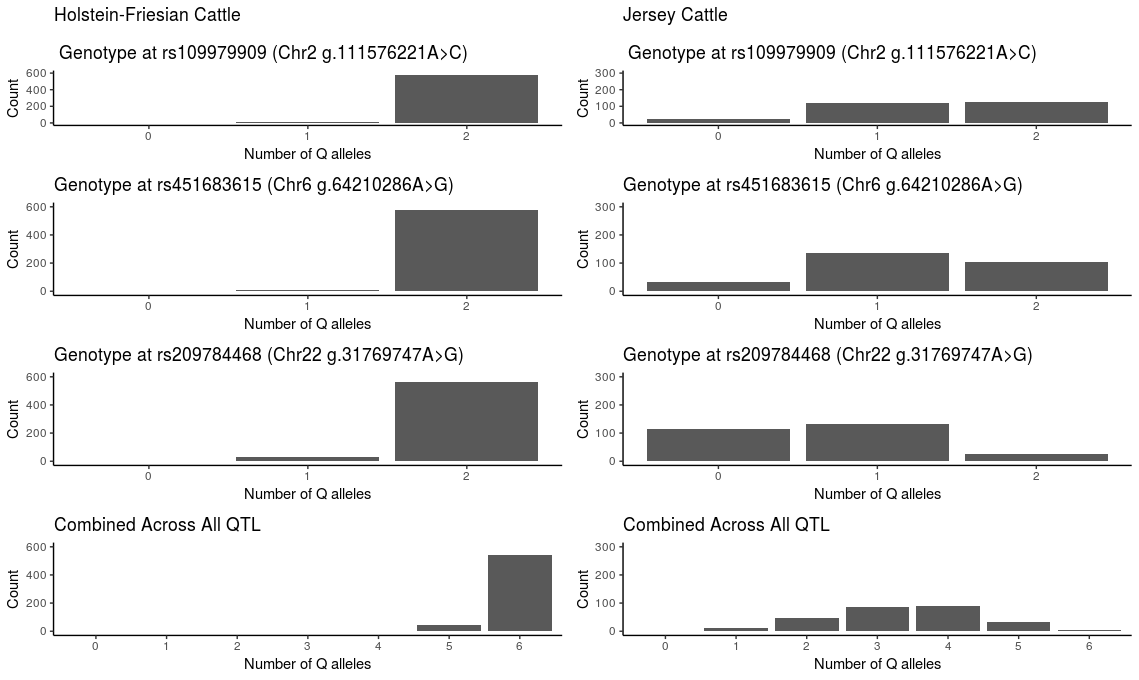


**Figure S3** Distribution of Q allele counts for each tag variant and combined across loci in cattle identified to be purebred Holstein-Friesian (left) and Jersey (right) within our mapping population.
